# Development of Silica Nanoparticle Supported Imprinted Polymers for Selective Lysozyme Recognition

**DOI:** 10.3390/nano11123287

**Published:** 2021-12-03

**Authors:** Anika Kotyrba, Mehmet Dinc, Boris Mizaikoff

**Affiliations:** 1 Institute of Analytical and Bioanalytical Chemistry, Ulm University, Albert-Einstein-Allee 11, 89081 Ulm, Germany; anika.kotyrba@uni-ulm.de; 2 Hahn-Schickard, Ulm, Sedanstraße 14, 89077 Ulm, Germany; mehmet.dinc@hahn-schickard.de

**Keywords:** molecularly imprinted polymers, MIP, protein imprinting, core-shell imprinting, core-shell particles, lysozyme

## Abstract

Protein imprinted MIPs show notable potential for applications in many analytical areas such as clinical analysis, medical diagnostics and environmental monitoring, but also in drug delivery scenarios. In this study, we present various modifications of two different synthesis routes to create imprinted core-shell particles serving as a synthetic recognition material for the protein hen egg white (HEW) lysozyme. HEW lysozyme is used as food additive E 1105 for preservation due to its antibacterial effects. For facilitating quality and regulatory control analysis in food matrices, it is necessary to apply suitable isolation methods as potentially provided by molecularly imprinted materials. The highest binding capacity achieved herein was 58.82 mg/g with imprinting factors ranging up to 2.74, rendering these materials exceptionally suitable for selectively isolating HEW lysozyme.

## 1. Introduction

Molecular imprinted polymers (MIPs) are crosslinked polymers with specific binding moieties for selectively binding target molecules. The interaction between the MIP and the corresponding target molecule is mimicking nature via various complementary binding processes (lock and key model), e.g., antigen-antibody, DNA-protein, ligand-receptor, and peptide–protein interaction. Therefore, imprinted polymers are expected to show a high affinity and selectivity towards the target molecule and should be able to discriminate and isolate them from closely related structures. MIPs can be prepared for a wide variety of targets, e.g., proteins, viruses, drugs or pesticides [1,2,3,4]. In contrast to natural receptors, MIPs can withstand much harsher conditions, such as high temperature, pressure, pH and organic solvents. Additionally, synthetic polymeric receptors are also less expensive to synthesize and their preparation can be easily scaled up for commercial and industrial applications [4]. The technology of molecular imprinting enables the creation of artificial binding sites within a polymer matrix. These sites are tailored in situ via co-polymerization of functional monomers and crosslinkers around the template [5,6]. Afterwards, the template molecule is extracted from the obtained polymer, leaving complementary cavities (regarding shape, size, and distribution of functional groups) within the polymeric network [1,2,3,4]. For this reason, MIPs show notable potential for applications in many analytical areas, such as clinical analysis, medical diagnostics, environmental monitoring, but more recently also for assisting drug delivery [4,7]. For example, they can be employed in high-performance liquid chromatography [8], solid phase extraction [9], sensors [10], separation [11,12], and catalysis [13,14]. However, the rebinding process depends on the diffusion of the template on the recognition sites. Therefore, small molecules are easier to imprint than macromolecules or entire biological species (i.e., peptides, proteins, DNA, viruses and bacteria), which can be limited by size, mass transfer, conformational instability, etc. [15] Specifically, mass transfer to and through the crosslinked polymer matrix is limited due to the molecular weight, which limits template removal and fast rebinding, which also results in a low binding capacity [15,16,17]. Because of this issue, MIPs prepared using the common bulky polymerization technique are not useful for proteins and other macromolecules [5]. Moreover, the protein molecules can be trapped deeply in the polymer matrix, which is resulting in a deteriorated elution [18,19]. Surface imprinting via nanoscale carrier materials is well suited for proteins as a template, considering the mentioned problems [15]. Moreover, the conditions here can be adapted more to the properties of the proteins. In the present study, core-shell imprinting was selected, which is a special application of the surface imprinting technique. Commonly used support materials for surface imprinting technique are TiO_2_, [20] Fe_2_O_3_, [21] and carbonaceous materials [22]. However, SiO_2_ was the most frequently used solid support material reported by numerous studies [5]. However, protein imprinting is still in the early stage, mainly because of the poor stability of the template protein during the polymerization, their limited solubility and stability (i.e., pH, temperature, etc.), as well as structural flexibility in solution [23].

Because biological macromolecules show a poor solubility and tend to change their conformational structure with their loss of biological activity in organic solvents, the imprinting is usually performed in aqueous environments. Due to the fact that many functional monomers are better soluble in organic solvents, the number of suitable monomers is limited [15].

The protein used as a template in our study was the Hen egg white (HEW) lysozyme. HEW lysozyme consists of 129 amino acids, with a molar mass of 14.3 kDa. It is a single peptide chain protein and contains 4 pairs of cysteines on the molecule forming 4 S-S bonds. The lysozyme is elliptical and has a size of 4.5 nm × 3.0 nm × 3.0 nm. The isoelectric point is at pI 11.35. Lysozyme exhibits a globular structure composed of four α-helices and five β-sheets, with four disulfide bridges [24]. The enzyme is active over a wide pH range (6.0 to 9.0). Maximum activity is observed at pH 6.2 [25,26,27,28]. The internal structure of lysozyme is almost non-polar, and hydrophobic interactions play an important role in the folded conformation of lysozyme [25,27]. Lysozymes preferentially hydrolyze the β-1,4-glycosidic bond between N-acetyl-D-muramic acid and 2-acetylamino-2-deoxy-D-glucose (=N-acetyl-D-glucosamine) in the sugar chains of the peptidoglucan scaffold of the bacterial cell wall. As a result, these enzymes have antibacterial properties. Lysozyme is abundant in a number of biological secretions, such as saliva, tears, sweat, human milk, earwax, and nasal and intestinal mucosa, as well as in blood plasma. Large amounts of lysozyme are found in hen egg white. In food industries, HEW lysozyme is approved within the EU as a food additive (i.e., number E 1105). For example, it is used at concentrations of up to 50 g/hl in winemaking to control acid biodegradation (i.e., bacterial breakdown of malic acid into lactic acid). Furthermore, it is used as a conservation agent for aged cheese and to preserve beer that has not been pasteurized or sterile-filtered [25,26,27]. For these reasons, it is necessary to be able to isolate and purify lysozyme. A common method for this so far is pH precipitation (salting out) and via chromatography on a cation exchange column. The salting-out process, based on the addition of a neutral salt, compresses the solvation layer and increases protein–protein interactions. When the salt concentration of a solution is enhanced, the charges on the surface of the protein interact with the salt, not with the water, exposing hydrophobic regions on the protein surface and causing the protein to precipitate out of solution [29]. The isoelectric point (pI) also plays an important role in this process. If the pH conforms to the pI, the protein has no netto charge. The negative and positive charges balance each other out, which reduces the repulsive electrostatic forces so that the attractive forces prevail. The attractive forces lead to aggregation and precipitation [29]. Thus, the solubility is the lowest at or close to this point. Lysozyme has a minimum solubility at around pH 10 [29]. This state of precipitation can be partially irreversible, so that the protein denatures. Moreover, the high pH can also lead to denaturation. This point is a disadvantage of this method [29]. Furthermore, the cation exchange chromatography works with the pI of the proteins. It can separate molecules based on their netto surface charge [30]. For this device, it is important that the target molecule, in this case lysozyme, has a positive surface charge. Thus, a pH of below 9 is required. This method involves several steps: the buffer preparation, the column equilibration, the sample loading, the column washing and the elution of the protein [30,31]. This involves a certain amount of effort. Furthermore, the effectiveness of this method is also highly pH dependent, if the pH is too close to the pI the amount of positive charges decreases. Moreover, to separate several proteins from each other using this method a salt gradient is then used to separate the protein of interest from other bound proteins, so they will be eluted in an order depending on their net surface charge [30,31]. This requires knowledge about their pIs. These points make this method more sophisticated in its usage. MIPs have the potential to represent an alternative, inexpensive method for lysozyme purification.

## 2. Materials and Methods

### 2.1. Chemicals

Acrylamide (AAM; ≥99%; Product No. A3553), Ammonium persulfate (APS; 98%; Product No. 215589), Hexadecyltrimethylammonium bromide (CTAB; ≥99%; Product No. H6269), Lysozyme from chicken egg white (HEW Lyz; ≥90%; Product No. L6876), Methacrylic acid (MAA; 99%; Product No. 155721), *N,N′*-Methylenbisacrylamide (MBA; 99%; Product No. 146072), *N,N,N′,N′*-Tetramethylethylendiamine (TEMED; 99%; Product No. T22500), Sodium Citrate tribasic dihydrate (≥99%; Product No. 71405), Thermolysin from Geobacillus stearothermophilus (Therm; Product No. T7902) and Trypsin from bovine pancreas (Tryp; Product No. T8003) were purchased from Sigma Aldrich (Steinheim, Germany). (3-Aminopropyl)-triethoxysilane, (APTES; 98%; Product No. A10668), (3-Aminopropyl)-trimethoxysilane (APTMS; 97%; Product No. A11284) and Hydrochloric acid solution (HCl; 1M; Product No. 35640) were acquired by purchase from Alfa Aesar (Kandel, Germany). Ammonia solution (28–30%; Product No. 1.05423) and Ethanol (99.5%; Product No. 8.5033) were obtained from VWR International. Citric acid trisodium salt dihydrate (99%; Product No. A12274) was purchased from Acros organics (Geel, Belgium). 2-(Dimethylamino)-ethyl methacrylate (DMAEMA; ≥99%; Product No. 8.40083), Dimethylformamide (DMF; 99.9%; Product No. 1.02375), di-Sodium hydrogen phosphate 2-hydrate (≥99.5%; Product No. 1.06580), Glutaraldehyde (50% solution in water; Product No. 8.14393), Potassium Chloride (≥99.5%; Product No. 1.04933), Potassium di-hydrogen phosphate (≥99.0%; Product No. P0662), Sodium Chloride (≥99.5%; Product No. 1.06404) and Tetraethyl orthosilicate (≥99%; Product No. 8.00658) were purchased from Merck (Darmstadt, Germany).

### 2.2. Synthesis of Core-Shell MIP Particles

#### 2.2.1. Synthesis of the Silica Cores

Amino-functionalized silica (SiO_2_-NH_2_) cores were synthesized using Stöber synthesis. For this purpose, 30 mL of ammonia solution (28–30%) were mixed with 150 mL ethanol and stirred for 10 min. Simultaneously, a solution of 30 mL ethanol, 4 mL APTES and 2 mL TEOS was prepared and stirred for 5 min. Then, the solutions were combined and stirred for 30 min at 25 °C. This results in silica cores C1. For variation of the core properties, 25 mg Cetyltrimethylammoniumbromide (CTAB) were added to the mixture. However, these modified silica particles denoted as cores C2 were only used in synthesis route I. To finish the reaction, another 50 mL ethanol were added to the solution. The particles were collected by centrifugation (4200 rpm) and washed with ethanol. Finally, the particles were dried overnight in a vacuum oven at 40 °C and 600 mbar.

#### 2.2.2. Synthesis Route I (Organic Shell, OC)

Functionalization of the core surface: To introduce polymerizable double bonds and terminal carboxyl groups onto the core surface of the SiO_2_-NH_2_, maleic anhydride was used. For this, the SiO_2_-NH_2_ cores (250 mg) were dispersed in 12 mL DMF by ultrasonic. Subsequently, maleic anhydride (315 mg) and pyridine (210 µL) were added, and the mixture was stirred for 3 h at 80 °C. The particles were collected by centrifugation, washed with ethanol and dried overnight (40 °C and 600 mbar). These particles are denoted as SiO_2_-COOH.

Imprinting Step: The crosslinking degree of this route is 50% and the molar ratio of AAM/MAA/DMAEMA is 1/0.12/0.12. The procedure of imprinting was carried out as described below. The SiO_2_-COOH particles (120 mg) were dispersed in 35 mL PBS (pH = 7.4) or citrate buffer (pH = 6.2). Then, MBA (80 mg), AAM (57.4 mg), MAA (8 mg), DMAEMA (14.6 mg) and Lyz (32 mg) were added to the suspension. The mixture was shaken for 1 h to obtain the pre-polymerization complex. As a next step, the mixture was purged with argon for 20 min to remove oxygen from solution. By injecting 120 µL APS solution (10 or 20%, (*w*/*v*)) and 60 µL of TEMED solution (5%, (*v*/*v*)), the polymerization reaction was initiated. The mixture was stirred at 25 °C for 24 h. The obtained particles were collected with centrifugation (2800 rpm) and washed two times with deionized water in order to remove oligomers and unreacted monomers. The obtained particles were washed repeatedly with 0.5 M NaCl solution for template extraction until no Lyz was detected in the supernatant at 280 nm. After re-washing with deionized water to remove the remaining NaCl, the particles were dried overnight (40 °C and 600 mbar). Control polymers (NIPs) were prepared by the same synthesis protocol but in the absence of lysozyme. These core-shell particles have an inorganic (silica) core and an organic shell.

#### 2.2.3. Synthesis Route II (Inorganic Shell, IC)

Functionalization of the core surface: In this route, no linker was required for grafting the polymer layer onto the core surface as the monomers and crosslinkers can be attached directly to the surface. However, to immobilize and orient the lysozyme prior to polymerization, the core particles were functionalized with glutaraldehyde. First, the SiO_2_-NH_2_ particles (300 mg) were dispersed in 12 mL water using ultrasonic. Then, a surplus of glutaraldehyde (165 µL, 7 µmol/mg particles) was added to the suspension and the mixture was shaken for 30 min at 25 °C. Afterwards, the particles were washed with deionized water and subsequently dried overnight (40 °C and 600 mbar).

Imprinting step: For the imprinting, the functionalized particles (250 mg) were suspended in 20 mL PBS (pH = 7.4) or citrate buffer (pH = 6.2). To the particles, 20 mL of lysozyme solution (2 mg/mL) were added. The particles were incubated with the lysozyme solution by shaking for 1 h. After removing the lysozyme solution, another 20 mL PBS or citrate buffer were added to the particles. Moreover, 4 µL APTES, 4 µL APTMS and 25 µL TEOS were added and the mixture was shaken for 24 h. Control polymers were prepared in the same way, but without the incubation step. These core-shell particles have an inorganic (silica) core and an inorganic (silica) shell.

Table 1 provides an overview to the different synthesis variations and their abbreviations.

In total, 5 variants of synthesis route I (OC) were successfully synthesized. Of these, 3 had silica particles C1 as the core (OC1), while 2 variants owned silica particles C2 as the core (OC2). Furthermore, 2 variants of synthesis route II were prepared (IC), both using silica particles C1 as core.

### 2.3. Protein Rebinding Experiments

For the investigation of the obtained rebinding properties (binding kinetics, binding capacity and selectivity) of the core-shell particles, incubation rebinding experiments were examined. For this purpose, 10 mg of dried particles were suspended in 750 µL of 0.5–1.0 mg/mL protein solutions prepared using PBS (pH = 7.4) or citrate buffer (pH = 6.2) as a solvent. The standard incubation time was 30 min. Competitive binding studies testing selectivity using trypsin and thermolysin were performed in the same way using a protein solution with the concentration 1.0 mg/mL. All studies were performed at room temperature. The amount of adsorbed protein by the nanoparticles after the rebinding experiments was calculated using the following formula:

$$q=\frac{\left(c_{i}-c_{f}\right)\cdot \;V}{m}$$

where q (mg/g) is the mass of protein adsorbed by unit mass of dry particles, *c_i_* (mg/mL) and *c_f_* (mg/mL) are the protein concentrations of the initial and the final solutions. *V* (mL) is the total volume of the mixture, while *m* is the mass of the applied particles.

### 2.4. Particle Characterization

Scanning electron microscopy (SEM) images were taken on a Quanta 3D FEG (FEI Company USA, Hillsboro, OR, USA) operated at 3.00 kV. The Brunauer–Emmett–Teller (BET) measurements were performed on a Quatrasorb SI/Quarasorb evo (Quantachrome/3P-Instruments, Odelzhausen, Germany). The Dynamic Light Scattering (DLS) experiments were carried out on a Zetasizer Nano ZS (Malvern Instruments, Malvern, UK). The laser has a wavelength of 633 nm and the measurements were taken at an angle of 173°. All UV-Vis measurements were taken on a Specord S600 (Analytik Jena, Jena, Germany) at 280 nm.

## 3. Results

### 3.1. DLS Studies

Table 2 summarizes the results of the DLS experiments.

Table 2 shows that cores C1 have a smaller diameter compared to cores C2, i.e., the addition of CTAB leads to an increase in diameter. Both core particle types have a very small PdI and are nearly monodisperse. The DLS measurements confirm that a polymer layer has formed around the core particles as the diameter has increased in all cases. Furthermore, the particle diameters of the MIPs are always larger than those of the corresponding NIPs; thus, the polymer layer formed is thicker. It can also be seen that doubling the starter concentration leads to the formation of a thicker polymer layer (see OC1_10%_ and OC1_20%_). Comparing the OC1_20%_ and OC1_Citrate_ synthesis variants, it can be stated that the diameters are significantly smaller for OC1_Citrate_ as for OC1_20%_. Larger diameters were also obtained for the OC2 and IC variants in PBS rather than in citrate buffer. Thus, pH has an influence on the thickness of the polymer layer. Particles C1 also result in MIP/NIP pairs whose diameters sometimes diverge by a factor of 2. The MIP/NIP pairs of particles C2, on the other hand, have similar diameters.

### 3.2. BET Studies

Table 3 shows the results of the BET measurements.

From Table 3 it is evident that the surface properties of the bare silica particles are influenced by their composition. The deviating pore diameters are particularly noticeable here. However, depending on the pore diameter, not the entire surface is available for adsorption of lysozyme. As mentioned, the dimensions of lysozyme are 4.5 nm × 3.0 nm × 3.0 nm. Taking this fact into account, lysozyme can diffuse more easily into the pores of particles C1 than into the pores of cores C2. For all MIPs and NIPs which have silica particles C1 as their core, it can be established that the pore diameters were significantly decreased (factor 3–4). On the basis of this fact, it can be assumed that polymer has built up in the pores resulting in a reduced pore diameter. For both cores C1 and C2, the surface areas increased after the imprinting step (with exception of NIP(IC_PBS_), revealing a slight decrease). Since there is a change in surface properties for all variations, it can be assumed that a polymer layer has formed at the particles.

### 3.3. SEM Studies

**Figure 1:**
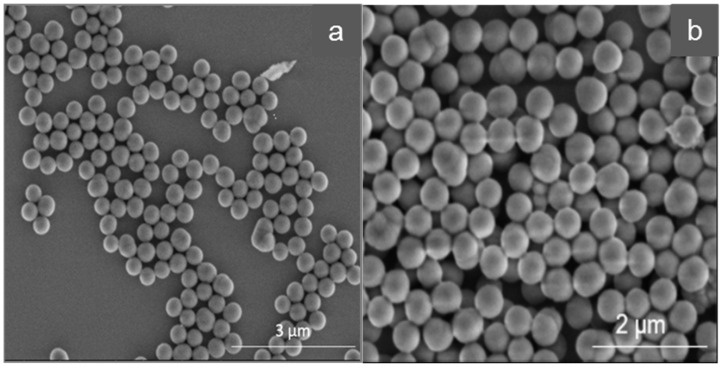
Comparison of the silica core particles (SEM images), (**a**): cores C1, (**b**): cores C2.

Figure 1 provides a comparison between the core particles C1 and C2.

Particles C1 are spherical, with a narrow size distribution. Their average size is 330.60 nm (see Table 2). The particles are non-agglomerated and have no visible pores. Based on the BET measurements (see Table 3), it is evident that these particles have small pores with an average diameter of 12.88 nm. Particles C2 appear slightly more inhomogeneous in shape, but otherwise there are no visible differences to particles C1. Their average size is 416.50 nm (see Table 2) and they have also a narrow size distribution. These particles also have no evident pores with the determined pore diameter averaging 3.87 nm (compare Table 3).

**Figure 2:**
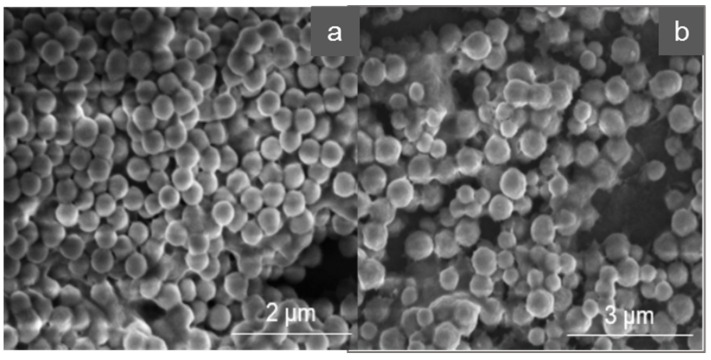
SEM images of MIP (**a**) and NIP (**b**); variant OC1_Citrate_.

Figure 2 shows exemplary the SEM images of MIP and NIP OC1_Citrate_.

Compared to the initial particles C1 (see Figure 1), the surface of MIP and NIP appears significantly modified. The surface of the core-shell polymers is corrugated and it is evident that a polymer coating has been formed both at the surface and between particles. In addition, MIP and NIP have a larger diameter than the initial particles C1. The modification of the surface was verified via DLS and BET measurements (compare Table 2 and Table 3). The MIPs are slightly more agglomerated than the NIPs.

### 3.4. Synthesis Route I (OC)

**Figure 3:**
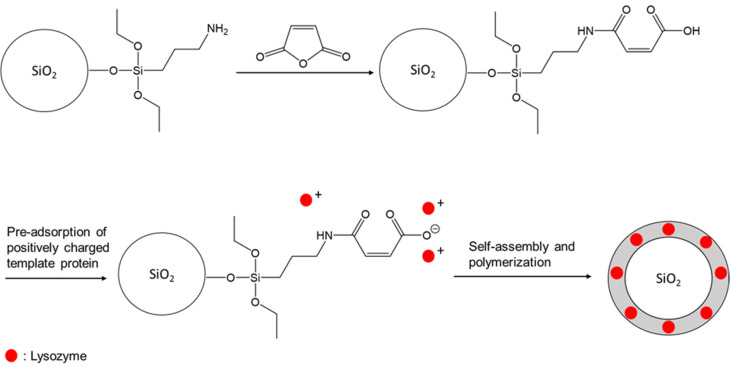
Synthesis route I (organic polymeric shell).

A schematic representation of synthesis route I is depicted in Figure 3.

#### 3.4.1. Polymers OC1_10%_

To gain an insight into binding kinetics, rebinding studies were carried out at incubation times of 30 min, 60 min and 240 min. Thereby, 1.0 mg/mL was chosen as initial the lysozyme concentration. The study was performed in PBS. Table 4 shows the results.

Table 4 shows that only the incubation times of 30 min and 60 min result in an imprinting factor higher than 1. It is also noticeable that Q_MIP_ and imprinting factor continue to decrease with increasing incubation time and the best values are obtained after only 30 min. Thus, if the incubation period is too long, the number of non-specific bindings increases and outweighs the specific ones, resulting in a lower imprinting factor. Therefore, 30 min was chosen as the standard incubation time.

To analyze the influence of the lysozyme concentration on the binding properties, a concentration-dependent study was performed in PBS.

Table 5 summarizes the results of this study.

Q_MIP_ rise with increasing lysozyme concentration with a rapid gain between 0.75 mg/mL and 1.0 mg/mL. All imprinting factors are larger than 1, the highest imprinting factor is obtained at the initial concentration of 0.75 mg/mL. Nonspecific binding is assumed to be the reason for the decrease to higher concentrations.

#### 3.4.2. Polymers OC1_20%_

As the next step, the concentration of the initiator (APS solution) was doubled, resulting in variant OC1_20%_. With these polymers, concentration-dependent rebinding studies were performed in PBS and citrate buffer to check the influence of both lysozyme concentration and pH during the rebinding process.

Table 6 summarizes the results for the concentration-dependent rebinding studies.

Comparing the results in PBS with those from variant OC1_10%_, Q_MIP_ increased significantly for the lysozyme concentrations 0.5 mg/mL and 0.75 mg/mL, while it decreased slightly for 1.0 mg/mL (compare with Table 5). For 0.5 mg/mL, the imprinting factor increased in this synthesis variant, while the opposite can be observed for the higher concentrations.

Both for PBS and citrate buffer, Q_MIP_ increases with the increasing lysozyme concentration, while the imprinting factors follow the opposite trend. Therefore, it is evident that the specific binding sites are already largely occupied at lower concentrations, and non-specific bonds are formed at higher concentrations. However, their influence is in general less pronounced in citrate buffer than in PBS. It may be observed that the specific binding capacities Q_MIP_ are higher in citrate buffer than in PBS; these observations could be due to the fact that this pH corresponds to the point of maximum activity of the lysozyme.

Thus, in direct comparison, pH = 6.2 proves to be more advantageous, at least for this synthesis variant.

**Figure 4:**
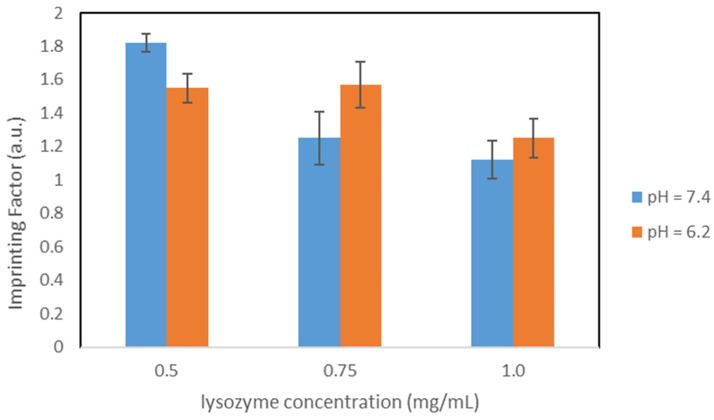
Comparison of the IFs, particles OC1_20%_.

A graphical comparison of the imprinting factors in both buffers is shown in Figure 4.

In order to verify the selectivity of the polymers, a comparative study was carried out in citrate buffer. The results are shown further down.

#### 3.4.3. Polymers OC1_Citrate_

To determine whether the pH during the synthesis has an influence on the binding quality of the polymers, citrate buffer was investigated as solvent instead of PBS resulting in synthesis variation OC1_Citrate_. All other synthesis parameters correspond to synthesis variant OC1_20%_. The results of the concentration dependent study in citrate buffer are shown in Table 7.

**Figure 5:**
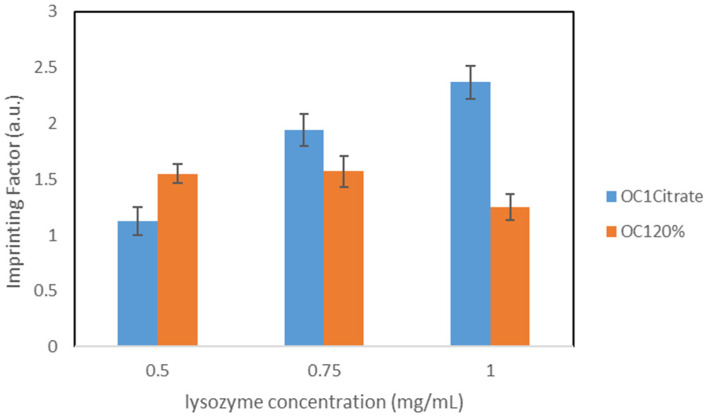
IFs for Particles OC1_20%_ and OC1_Citrate_, pH = 6.2.

The results conclude that Q_MIP_ correlate with the initial concentration, i.e., the higher the initial lysozyme concentration, the higher the Q_MIP_. The same trend can be observed for the imprinting factors. Since neither Q_MIP_ nor imprinting factors passed through a maximum, the particles are still specific even at higher lysozyme concentrations. Furthermore, it can be deduced that many specific binding sites were formed that were not yet completely occupied even at 1.0 mg/mL. Compared with the same study of the analog synthesis variant OC1_20%_ (see Table 6), this variant provides better binding properties. Thus, higher Q_MIP_ was achieved at all concentrations. A graphical comparison of the imprinting factors of polymers OC1_20%_ and OC1_Citrate_ in citrate buffer can be seen in Figure 5.

With the exception of 0.5 mg/mL, the imprinting factors rise using polymers OC1_Citrate_. This optimization can be attributed to the fact that the pH during synthesis corresponds to the optimum of lysozyme activity. This synthesis variant provides a promising approach with respect to the objective of this work. Moreover, good binding characteristics are observed here even at high lysozyme concentrations.

Likewise, a competitive study was performed in citrate buffer, and the results are presented in a latter chapter.

#### 3.4.4. Polymers OC2_PBS_ and OC2_Citrate_

To gain an insight into the influence of the silica core on the binding properties, the silica core was modified by adding 25 mg CTAB during the Stöber synthesis. This results in the OC2 variants. All other synthesis parameters were retained. Both buffers were tested during the synthesis.

##### Polymers OC2_PBS_

The results for the concentration dependent studies both in PBS and citrate buffer are shown in Table 8.

In PBS, both IF and Q_MIP_ follow the same trend: a maximum can be observed at the concentration 0.75 mg/mL. If this study is now compared with the analogous study with the polymers OC1_20%_ (identical synthesis, different silica core), better results are obtained (compare Table 6). Q_MIP_ of the OC2_PBS_ polymers are higher in all concentrations. Similarly, this variant has better imprinting factors at high lysozyme concentrations. At low concentrations, on the other hand, the OC1_20%_ variant is preferable, since the imprinting factor here is significantly higher and thus the proportion of non-specific binding is smaller. In citrate buffer, on the other hand, the imprinting factor is highest for the initial lysozyme concentration of 1.0 mg/mL. The imprinting factors do not behave proportionally to the initial concentrations, a minimum can be seen at 0.75 mL/mL. Q_MIP_ grows with increasing concentration, and the highest value was received for 1.0 mg/mL.

**Figure 6:**
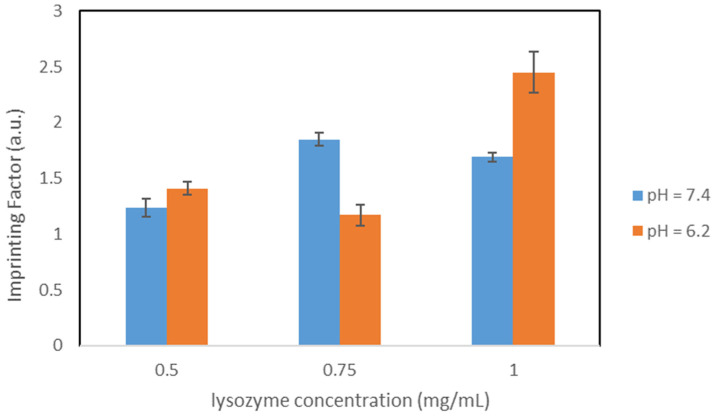
Comparison of the IFs, particles OC2_PBS_.

The highest Q_MIP_ and imprinting factor for the OC2_PBS_ variant were both obtained in citrate buffer for a lysozyme concentration of 1.0 mg/mL. Figure 6 compares the concentration dependent IFs during both pH values.

Comparing both buffers for variant OC2_PBS_, it can be stated that citrate buffer is better qualified for higher lysozyme concentrations. PBS tends to be better suited for lower lysozyme concentrations, since higher Q_MIP_ were obtained here.

Comparing this study in citrate buffer with the analogous study with the particles OC1_20%_ (see Table 6) can be established that Q_MIP_ increases for all concentrations. The opposite trend can be observed for the IFs (for the concentrations 0.5 mg/mL and 0.75 mg/mL). For the concentration 1.0 mg/mL, on the other hand, the imprinting factor is almost doubled.

The results for the competitive study in citrate buffer can be found subsequent.

##### Polymers OC2_Citrate_

The results for the concentration dependent studies in PBS and citrate buffer are shown in Table 9.

For these polymers in PBS, both the highest imprinting factor and Q_MIP_ were obtained for the initial lysozyme concentration 0.75 mg/mL. However, the decrease in both quantities to the concentration 1.0 mg/mL is relatively minor. In contrast, significantly poorer binding properties were obtained for 0.5 mg/mL. These two aspects make this variant in PBS more applicable for higher lysozyme concentrations. Moreover, in citrate buffer, the highest imprinting factor was obtained at 0.75 mg/mL, while the highest Q_MIP_ is obtained for 1.0 mg/mL. The imprinting factors decreases sharply between 0.75 mg/mL and 1.0 mg/mL indicating an increase in non-specific binding to high concentrations.

**Figure 7:**
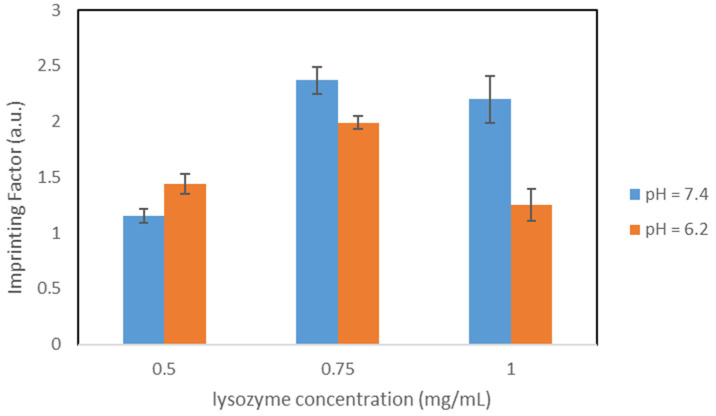
Comparison of the IFs, particles OC2_Citrate_.

Figure 7 compares the imprinting factors of particles OC2_Citrate_ in both buffers.

However, higher imprinting factors were obtained for 0.75 mg/mL and 1.0 mg/mL in PBS rather than in citrate buffer. For 0.5 mg/mL, on the other hand, an increase in both Q_MIP_ and imprinting factor is observed in citrate buffer.

When the results in citrate buffer are compared with the analogous study with particles OC1_Citrate_ (see Table 7), it is shown that the imprinting factors for the two smaller concentrations have increased, while the imprinting factor for 1.0 mg/mL has nearly halved. For the two higher concentrations, Q_MIP_ decreased compared to the OC1_Citrate_ variant.

Table 10 compiles the results of the completed studies.

It is noticeable that the target protein lysozyme binds better to the OC1_20%_ MIP than the reference proteins. Thus, it can be concluded that the polymer particles show a degree of selectivity in citrate buffer.

For the OC1_Citrate_ variant, the MIP binds the lysozyme better than thermolysin, and so there is a selectivity towards thermolysin. However, trypsin is bound slightly better by particles OC1_Citrate_ than by lysozyme. It is noteworthy that the ratio of the binding capacities between MIP and NIP is more pronounced for lysozyme than for both reference proteins. For the OC2_PBS_ version it applies that trypsin is best bound by MIP, but the difference with lysozyme is negligible. Thermolysin is less bound by the MIPs. For OC_Citrate_, Q_MIP_ is significantly larger for lysozyme than for trypsin and thermolysin. The particles are thus selective.

### 3.5. Synthesis Route II (IC)

**Figure 8:**
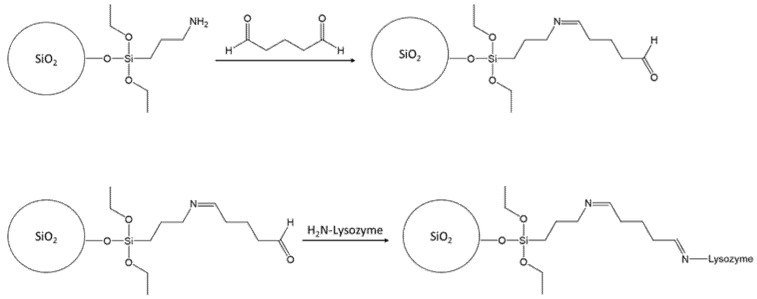
Synthesis route II (inorganic silica shell).

A schematic representation of synthesis route II is depicted in Figure 8.

This synthesis was also performed in PBS and citrate buffer.

#### 3.5.1. Polymers IC_PBS_

The results for the concentration dependent study in PBS are shown in Table 11.

In PBS, both Q_MIP_ and imprinting factors increase with increasing lysozyme concentration, no maximum was reached for both parameters in the investigated concentration range. Thus, the non-specific binding does not have a considerable influence here.

As in PBS, Q_MIP_ rises in citrate buffer with increasing lysozyme concentration, but reaches higher values. However, since the imprinting factors decrease with increasing lysozyme concentration, this gain can be attributed to non-specific binding.

**Figure 9:**
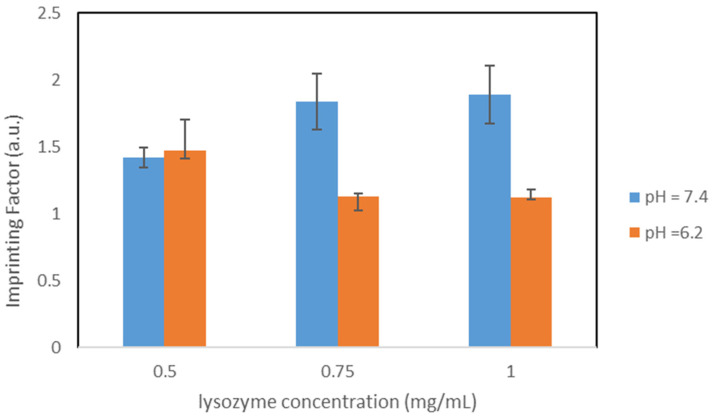
Comparison of the IFs, particles IC_PBS_.

Figure 9 represents the IF for the different concentrations for both pH values.

The highest imprinting factor for these particles is obtained in PBS at lysozyme concentration 1.0 mg/mL. For smaller lysozyme concentrations, it is thus advantageous to use the polymers in citrate buffer, since the imprinting factor and Q_MIP_ are higher here. For higher concentrations (0.75 mg/mL and 1.0 mg/mL), PBS is better suited.

#### 3.5.2. Polymers IC_Citrate_

Table 12 shows the results of the concentration-dependent study in PBS.

In PBS, Q_MIP_ grows with increasing lysozyme concentration. However, the highest imprinting factor is obtained for the lysozyme concentration 0.75 mg/mL, so the proportion of non-specific binding increases at higher concentrations. Comparing this study with the analog synthesis (particles IC_PBS_ in PBS, see Table 13) it can be noted that Q_MIP_ has increased for all concentrations. The opposite can be observed for the IF. This leads to the conclusion that the growth of Q_MIP_ is due to a high proportion of non-specific binding.

**Figure 10:**
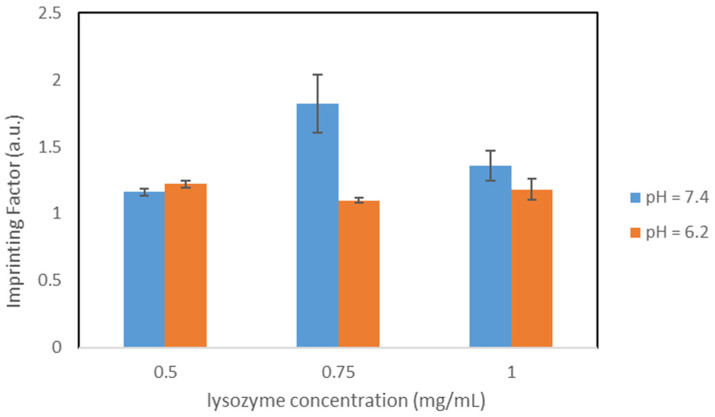
Comparison of the IFs, particles IC_Citrate_.

Moreover, in citrate buffer, Q_MIP_ increases with growing lysozyme concentration; however, the highest imprinting factor is obtained for 0.5 mg/mL. A plot comparison of the obtained imprinting factors is shown in Figure 10.

The highest imprinting factor for these polymers is obtained in PBS at 0.75 mg/mL. At higher lysozyme concentrations higher Q_MIP_ and imprinting factors were received in PBS. Thus, PBS is more suitable for higher lysozyme concentrations.

Q_MIP_ of lysozyme is higher than for the reference proteins. However, the difference to trypsin is minimal. Thus, a degree of selectivity can be assumed. What is noticeable here is that the ratio of Q_MIP_ and Q_NIP_ for lysozyme is better than it is for the reference proteins.

In conclusion, Q_MIP_ has increased with increasing lysozyme concentration for both variants in both buffers.

Both IC_PBS_ and IC_Citrate_ exhibit higher IF in PBS than in citrate buffer at higher lysozyme concentrations (0.75 mg/mL and 1.0 mg/mL).

## 4. Conclusions

The present study provides an insight into the development of molecularly imprinted polymers for HEW lysozyme. Using the core-shell approach, specific synthetic receptors for the model protein were successfully generated. By adjusting the conditions, satisfactory imprinting efficiency with respect to both imprinting factors and binding capacities was achieved. Thus, this method represents a promising alternative to current methods for the isolation of lysozyme. Furthermore, the imprinted particles showed fast rebinding kinetics. As for selectivity, the highest imprinting factor of the core-shell polymers I was 2.74, and for core-shell polymers II 1.89. Thus, a higher sensitivity to lysozyme was achieved using route I. Furthermore, route I achieved better selectivity towards trypsin and thermolysin than route II. The highest Q_MIP_ obtained for route I was 55.32 mg/g (C1 core) and 58.82 mg/g (C2 core), respectively. The highest Q_MIP_ received for route II was 47.40 mg/g.

Thus, the purpose of this study (i.e., high Q_MIP_ and high IF for lysozyme) was maximized via route I vs. route II. Nevertheless, the obtained results for route II were also satisfactory providing slightly less, but still high binding capacities and distinct selectivity. Last, but not least, the latter synthesis route is experimentally easier and less time-consuming, which may be decisive parameters for a scaled-up synthesis required by industrial application scenarios. We compared the results with the work of Zhang and others, where an imprinting factor of 2.53 was achieved. As for route II, this polymer is a silica-based polymer.

## Figures and Tables

**Table 1:** Abbreviations of the syntheses.

Abbreviation	Synthesis Route	Core Type	APS Solution (*w*/*v*)	pH	Extraction
OC1_10%_	I	1	10%	7.4	0.5 M NaCl
OC1_20%_	I	1	20%	7.4	0.5 M NaCl
OC1_Citrate_	I	1	20%	6.2	0.5 M NaCl
OC2_PBS_	I	2	20%	7.4	0.5 M NaCl
OC2_Citrate_	I	2	20%	6.2	0.5 M NaCl
IC_PBS_	II	1	-	7.4	1 M HCl
IC_Citrate_	II	1	-	6.2	1 M HCl

**Table 2:** DLS Results.

Abbreviation	Z-Average d [nm]	PdI
C1	330.60	0.03
C2	416.50	0.01
MIP(OC1_10%_)	970.60	0.14
NIP(OC1_10%_)	609.37	0.04
MIP(OC1_20%_)	1203.33	0.29
NIP(OC1_20%_)	824.03	0.22
MIP(OC1_Citrate_)	539.33	0.11
NIP(OC1_Citrate_)	401.87	0.65
MIP(OC2_PBS_)	873.37	0.34
NIP(OC2_PBS_)	825.97	0.42
MIP(OC2_Citrate_)	481.20	0.13
NIP(OC2_Citrate_)	432.50	0.06
MIP(IC_PBS_)	1005.00	0.67
NIP(IC_PBS_)	497.37	0.14
MIP(IC_Citrate_)	707.30	0.87
NIP(IC_Citrate_)	341.40	0.28

**Table 3:** BET results.

Abbreviation	Surface Area (Adsorption) (m^2^/g)	Pore Diameter (Adsorption) (nm)	Pore Volume (Adsorption) (cc/g)
C1	12.16	12.88	0.06
C2	11.48	3.87	0.04
MIP(OC1_10%_)	13.87	4.66	0.05
NIP(OC1_10%_)	50.07	3.00	0.10
MIP(OC1_20%_)	34.48	3.54	0.08
NIP(OC1_20%_)	21.86	3.74	0.07
MIP(OC1_Citrate_)	18.14	3.01	0.04
NIP(OC1_Citrate_)	12.95	3.49	0.04
MIP(OC2_PBS_)	22.76	3.32	0.07
NIP(OC2_PBS_)	24.84	4.17	0.07
MIP(OC2_Citrate_)	41.81	5.77	0.09
NIP(OC2_Citrate_)	92.85	3.66	0.15
MIP(IC_PBS_)	28.73	3.51	0.06
NIP(IC_PBS_)	11.96	3.66	0.04
MIP(IC_Citrate_)	17.57	8.04	0.11
NIP(IC_Citrate_)	17.42	4.11	0.09

**Table 4:** Results of time dependent rebinding study of polymers OC1_10%_ in PBS.

Incubation Time (min)	Q_MIP_ (mg/g)	Q_NIP_ (mg/g)	IF (a.u.)
30	43.59	33.83	1.29
60	23.38	20.35	1.14
240	2.9	19.53	0.15

**Table 5:** Results of concentration dependent rebinding study of polymers OC1_10%_ in PBS.

Lysozyme Concentration (mg/mL)	Q_MIP_ (mg/g)	Q_NIP_ (mg/g)	IF (a.u.)
0.5	2.36	1.45	1.63
0.75	3.14	1.14	2.74
1.0	43.59	33.83	1.29

**Table 6:** Results of concentration dependent rebinding study of polymers OC1_20%_.

Lysozyme Concentration (mg/mL)	Q_MIP_ (mg/g)	Q_NIP_ (mg/g)	IF (a.u.)	pH
0.5	14.81	8.16	1.82	7.4
0.75	21.77	17.42	1.25	7.4
1.0	35.73	31.95	1.12	7.4
0.5	18.80	12.13	1.55	6.2
0.75	25.05	15.99	1.57	6.2
1.0	46.28	37.07	1.25	6.2

**Table 7:** Results of concentration dependent rebinding study of polymers OC1_Citrate_ in citrate buffer.

Lysozyme Concentration (mg/mL)	Q_MIP_ (mg/g)	Q_NIP_ (mg/g)	IF (a.u.)
0.5	24.36	21.73	1.12
0.75	42.93	22.09	1.94
1.0	55.32	23.35	2.37

**Table 8:** Results of concentration dependent rebinding study of polymers OC2_PBS_.

Lysozyme Concentration (mg/mL)	Q_MIP_ (mg/g)	Q_NIP_ (mg/g)	IF (a.u.)	pH
0.5	31.17	25.13	1.24	7.4
0.75	41.87	22.62	1.85	7.4
1.0	38.05	22.49	1.69	7.4
0.5	28.61	20.24	1.41	6.2
0.75	32.92	28.05	1.17	6.2
1.0	52.46	21.40	2.45	6.2

**Table 9:** Results of concentration dependent rebinding study of polymers OC2_Citrate_.

Lysozyme Concentration (mg/mL)	Q_MIP_ (mg/g)	Q_NIP_ (mg/g)	IF (a.u.)	pH
0.5	26.80	23.30	1.15	7.4
0.75	58.82	24.80	2.37	7.4
1.0	57.35	26.02	2.20	7.4
0.5	28.61	20.24	1.44	6.2
0.75	26.85	13.47	1.99	6.2
1.0	46.17	37.06	1.25	6.2

**Table 10:** Results of the comparative studies of polymers OC1_20%_, OC1_Citrate_, OC2_PBS_ and OC2_Citrate_ in citrate buffer.

Protein	Q_MIP_ (mg/g)	Q_NIP_ (mg/g)	Polymers	pH
Lysozyme	46.28	37.07	OC1_20%_	6.2
Trypsin	38.65	32.99	OC1_20%_	6.2
Thermolysin	31.92	20.79	OC1_20%_	6.2
Lysozyme	55.32	23.35	OC1_Citrate_	6.2
Trypsin	59.19	48.06	OC1_Citrate_	6.2
Thermolysin	37.94	30.26	OC1_Citrate_	6.2
Lysozyme	52.46	21.40	OC2_PBS_	6.2
Trypsin	52.95	37.89	OC2_PBS_	6.2
Thermolysin	41.10	23.53	OC2_PBS_	6.2
Lysozyme	46.17	37.06	OC2_Citrate_	6.2
Trypsin	25.87	11.08	OC2_Citrate_	6.2
Thermolysin	37.07	35.08	OC2_Citrate_	6.2

**Table 11:** Results of concentration dependent rebinding study of polymers IC_PBS_.

Lysozyme Concentration (mg/mL)	Q_MIP_ (mg/g)	Q_NIP_ (mg/g)	IF (a.u.)	pH
0.5	25.65	18.08	1.42	7.4
0.75	32.47	17.68	1.84	7.4
1.0	36.77	19.49	1.89	7.4
0.5	31.57	21.30	1.48	6.2
0.75	33.73	29.87	1.13	6.2
1.0	41.07	36.70	1.12	6.2

**Table 12:** Results of concentration dependent rebinding study of polymers IC_Citrate_ in PBS.

Lysozyme Concentration (mg/mL)	Q_MIP_ (mg/g)	Q_NIP_ (mg/g)	IF (a.u.)	pH
0.5	27.53	23.64	1.16	7.4
0.75	41.12	22.59	1.82	7.4
1.0	47.40	34.91	1.36	7.4
0.5	23.40	19.16	1.22	6.2
0.75	33.72	30.55	1.10	6.2
1.0	46.64	39.50	1.18	6.2

**Table 13:** Results of the comparative study of polymers IC_Citrate_ in PBS.

Protein	Q_MIP_ (mg/g)	Q_NIP_ (mg/g)	pH
Lysozyme	47.40	34.91	7.4
Trypsin	45.51	44.03	7.4
Thermolysin	39.14	32.16	7.4

## Data Availability

Data will be made available upon request.
